# Trade off between variable and fixed size normalization in orthogonal polynomials based iris recognition system

**DOI:** 10.1186/s40064-016-1909-y

**Published:** 2016-03-24

**Authors:** R. Krishnamoorthi, G. Anna Poorani

**Affiliations:** IRIS Laboratory, Department of Computer Science and Engineering, Bharathidasan Institute of Technology, Anna University, Tiruchirappalli, India

**Keywords:** Pupil border, Limbus border, Variable size normalization, Fixed size normalization

## Abstract

Iris normalization is an important stage in any iris biometric, as it has a propensity to trim down the consequences of iris distortion. To indemnify the variation in size of the iris owing to the action of stretching or enlarging the pupil in iris acquisition process and camera to eyeball distance, two normalization schemes has been proposed in this work. In the first method, the iris region of interest is normalized by converting the iris into the variable size rectangular model in order to avoid the under samples near the limbus border. In the second method, the iris region of interest is normalized by converting the iris region into a fixed size rectangular model in order to avoid the dimensional discrepancies between the eye images. The performance of the proposed normalization methods is evaluated with orthogonal polynomials based iris recognition in terms of FAR, FRR, GAR, CRR and EER.

## Background

Owing to differences in the image capturing distance and in the illuminationed environment, the size of the subject’s pupil and the iris region of interest in the captured images are highly diverging. An accumulation of this, there is discrepancies of the same eye images owing to stretches of the iris. Additional criteria that cause dilation are eye rotary motion, revolving camera and head inclination. Such a deformation of the iris texture broadens intra-class dissimilarities and raises the FRR.

A survey of the existing normalization techniques found in the literature is presented in the following section.

## Review of literature

From the perspective of iris texture feature extraction, the normalization techniques are classified into six categories such as Linear model (Daugman 1993; Lim et al. 2001; Joung et al. 2005; Boles and Boashash 1998; Ma et al. 2003; Subbarayudu and Prasad 2008; Shamsi and Rasouli 2009, 2011), variant of linear model (Krishnamoorthi et al. 2012), non-linear model (Wildes 1997; Wyatt 2000), the combination of non-linear and linear-models (Wei et al. 2007; Yuan and Shi 2005), non-polar coordinate normalization (Arvachech and Tizhoosh 2006) and irregular border normalization (Han et al. 2009; Shah and Ross 2009).

Daugman (1993) employed linear rubber sheet model which projects the doughnut shaped iris region of interest into a fixed rectangle region. Lim et al. (2001) have used a fixed resolution model very similar to the Daugman’s pseudo polar transform approach. They have normalized the distance between the pupil border and the limbus border into [0, 60] according to the arbitrary radius *r* and normalized the angular resolution into [0, 450] according to the step angle 0.8°. Joung et al. (2005) have unwrapped the iris with limbus center to define the polar coordinates of the points over the limbus border and used pupil center to define the polar coordinates of the pupil border. The coordinates of the other points between these two borders are obtained linearly in the radial direction. Boles and Boashash’s normalization technique (Boles and Boashash 1998) is also similar to Daugman’s method with the difference that it is performed at the time of matching. Their method is based on the virtual circles to map the iris features. Ma et al. (2003) have combined the Daugman’s method (Daugman 1993) and Bole’s method (Boles and Boashash 1998). They have used the pupil center as a reference point in their mapping strategy. Subbarayudu and Prasad (2008) have assumed that the pupil and limbus boundaries are two circles and utilized angular strips radial measure to map iris region. Shamsi and Rasouli (2009) have devised a new mapping strategy to rescale point. Shamsi and Rasouli (2011) have transformed iris disk to trapezium strip.

Krishnamoorthi et al. (2012) have devised a variation of trapezoidal model to avoid the under samples near the limbus border. Wildes (1997) has reported an image registration technique for compensating variations in rotation and scale. Wyatts et al. (2000) have used the virtual arc concept and carried out the mapping from the reference annular zone into a fixed-size rectangle zone. Wei et al. (2007) have utilized Gaussian function to estimate the additive variation of a nonlinear iris stretch. Yuan and Shi (2005) have considered the nonlinear behavior of iris patterns with a predefined ratio of the radii of the pupil and limbus boundaries of the iris. Arvachech and Tizhoosh (2006) have merged the non-linear model and linear model to unwrap an iris region of interest properly. Han et al. (2009) have designed a normalization method that does not adopt the polar coordinate transformation. They have preserved the original geometric structure and directional information. Shah and Ross (2009) have formulated the normalization technique for conical iris boundaries.

Motivated by the fact that iris boundaries are not in specific shapes, variable-size and fixed-size iris normalization techniques are proposed in this work for normalizing the irregular iris boundaries.

The important steps involved in the proposed normalization work are as follows:

- Estimation of the center and radius of pupil
- Estimation of the coarse radius of limbus
- Estimation of the accurate radius of the limbus
- Computation of the resolution angle of increment and
- Identification of the sampling points.

## Preprocessing

Initially, the coarse estimation of pupil center is found as the point that corresponds to local minima of image intensity. The extraction of coarse pupil localization area on four sides from the coarse pupil center is modeled with approximation of pupil center and radius to confine the search for the pupil border. Then, an edge image is generated by applying negatively sloped zero-crossing point with orthogonal polynomials (Ganesan and Bhattacharya 1997). The fine pupil boundaries are then extracted after detecting radial border points in the angular direction of the projection curve. The pupil border points are fitted using the cubic smoothing spline.

The limbus border extraction is then carried out with gradient based edge detection on the same orthogonal polynomials model. Initially, the coarse limbus region is estimated with approximation of pupil center and radius to confine the search for the limbus border. This coarse limbus region is subjected to the orthogonal polynomials and after that the precise limbus border points are extracted with vertical and horizontal edge detection. The limbus curvature is approximated with cubic smoothing spline from the limbus border points.

## Proposed variable-size normalization model

First let us consider the pupil and limbus border points present in localized image of the iris image. The radius of irregular pupil border is estimated from the pupil border points with the following steps. Along the *x*(minor) axis, the extreme positions at lower end (*X*_max_*x*_, *X*_max_*y*_) and higher end (*X*_min_*x*_, *X*_min_*y*_) are extracted from the pupil border points. Similarly for *y*(major) axis, the extreme positions at lower end (*Y*_max_*x*_, *Y*_max_*y*_) and higher end (*Y*_min_*x*_, *Y*_min_*y*_) are extracted from pupil border points. The distance, *x*_*dist*, between the extreme positions in *x* axis are computed as follows

1 $$x\_dist = \sqrt {\left( {X_{\hbox{max} \_x} - X_{\hbox{min} \_x} } \right)^{2} + \left( {X_{\hbox{max} \_y} - X_{\hbox{min} \_y} } \right)^{2} }$$

Similarly, the distance, *y*_*dist*, between the extreme positions in *y* axis are computed as follows

2 $$y\_dist = \sqrt {\left( {Y_{\hbox{max} \_x} - Y_{\hbox{min} \_x} } \right)^{2} + \left( {Y_{\hbox{max} \_y} - Y_{\hbox{min} \_y} } \right)^{2} }$$

In this work, the radius of the pupil border *r*_*p*_ is determined as

3 $$r_{p} = \frac{{\hbox{max} \left( {x\_dist,y\_dist} \right)}}{2}$$

The center of the pupil border, $\left( {x_{c}^{p} ,y_{c}^{p} } \right)$, is calculated with the relation

4 $$\left( {x_{c}^{p} ,y_{c}^{p} } \right) = \left( {\frac{{X_{\hbox{max} \_x} + X_{\hbox{min} \_x} }}{2},\frac{{Y_{\hbox{max} \_y} + Y_{\hbox{min} \_y} }}{2}} \right)$$

**Fig. 1:**
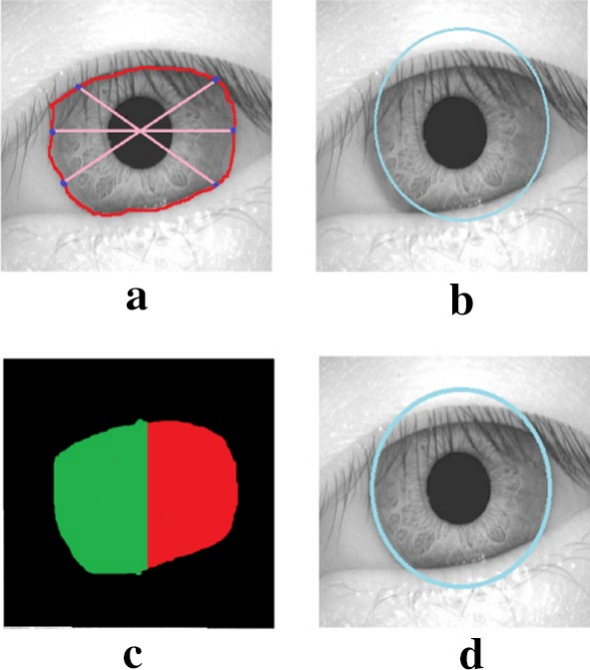
Estimation of limbus radius. **a** Points on the extracted contour. **b** Approximate limbus boundary. **c** Unequal area under the segmented limbus curve on either side of the vertical axis. **d** Accurate limbus boundary

After determining the coarse radius of pupil border, the radius of limbus border is calculated for iris normalization. If the obstruction owing to either the upper or lower eyelids is significant, in that case the circle that fits every point on the extorted contour will be positioned within the exact border of the limbus. For this reason, merely the points resting on the border of the limbus are modeled in this research work to calculate the radius and center of the limbus. To guarantee this, six points at the following angles [−30°, 0°, 30°, 150°, 180°, 210°] are chosen from the extorted contour as well as their mean distance from the center of the pupil is calculated and exploited as the coarse radius of the limbus (*r*_*l*_). A circle is subsequently fitted throughout every point on the contour that are within a distance of (*r*_*l*_ ± 10) pixels from the center of the pupil $\left( {x_{c}^{p} ,y_{c}^{p} } \right)$. The center and radius of such a circle is primarily modeled to be the center $\left( {x_{c}^{l} ,y_{c}^{l} } \right)$ and the radius (*r*_*l*_) of the limbus. When the limbus is detected in the corner of the eye, all six points preferred to calculate the rough limbus radius may not be positioned on the limbus border; some may be positioned on the eyelid. As the eyelid border is nearer to the center of the pupil, an approximate limbus radius is off-center and the same is demonstrated in Fig. 1a, b. Also the area beneath the segmented limbus curvature on either side of the perpendicular axis passing through the pupil is not uniform, is shown in Fig. 1c. The region with smaller area is heavily obstructed by the eyelids. Hence, if the differences among the two regions exceed 10 %, merely three points resting on the contour related to the bigger region are preferred to calculate the limbus radius. This gives a superior estimate of the limbus radius (*r*_*l*_) and it increases the accuracy even in the case of off-angle iris.

The maximum degree (360°) is divided into small units of angle increment factor that enables to reach each limbus border positions of the iris. The circumference $\varpi$ of the limbus border is calculated as

5 $$\varpi = 2\pi r_{l}$$

The distance between the pupil border and limbus border is normalized to [0, 360]. The resolution angle of increment $\phi$ is computed as

6 $$\phi = \frac{{360^{ \circ } }}{\varpi }$$

By increasing the angle *θ* by $\phi$ for radius *r*_*l*_, the variable-size rectangular resolution iris image for the plane (*θ*, *r*_*l*_) is obtained. Also the degree of rotation (360°) is calibrated in such a way that it reaches each position in the limbus border.

After determining the resolution angle of increment, the sampling points are extracted by tracing points from limbus border to pupil border. Let us initialize *θ* as 0, *x*_*i*_ as $x_{c}^{p}$, and *y*_*i*_ as $y_{c}^{p}$. Let the number of tracing points be *n* from limbus border to pupil border with the length $\left( {r_{l} - r_{p} } \right)$. Consider (*x*_*s*_, *y*_*s*_) and (*x*_*e*_, *y*_*e*_) as start and end positions respectively of a line identity and traverse along the line and obtain each position co-ordinates in the iris Region of Interest (ROI) by decrementing *n* by 1. The starting point (*x*_*s*_, *y*_*s*_) of tracing process is computed as follows

7 $$x_{s} = x_{i} + r_{l} Cos\left( {\frac{\pi \theta }{180}} \right)$$

8 $$y_{s} = y_{i} + r_{l} Sin\left( {\frac{\pi \theta }{180}} \right)$$

The end point (*x*_*e*_, *y*_*e*_) of tracing process is computed as

9 $$x_{e} = x_{i} + r_{p} Cos\left( {\frac{\pi \theta }{180}} \right)$$

10 $$y_{e} = y_{i} + r_{p} Sin\left( {\frac{\pi \theta }{180}} \right)$$

The *x*(minor) axis width is computed as

11 $$d_{x} = x_{e} - x_{s}$$

The *y*(major) axis width is computed as

12 $$d_{y} = y_{e} - y_{s}$$

The initial point in the limbus border is stored in angular resolution array *R*[*n*][*θ*]. Consider (*x*_*s*_, *y*_*s*_) and (*x*_*e*_, *y*_*e*_) as start and end positions respectively of a line identity and traverse along the line and obtain each position co-ordinates in the iris ROI by decrementing *n* by 1. If the absolute value of *d*_*x*_ is larger than *d*_*y*_, the point *R*[*n*][*θ*] of the slope *m* and y_intercept *b* is computed using the Eqs. (13) through (16).

13 $$m = \frac{{d_{y} }}{{d_{x} }}$$

14 $$b = y_{s} - m*x_{s}$$

The b (y_intercept) is computed as

15 $$If\left( {d_{x} < 0} \right)\quad then,\;\;d_{x} = - 1;\quad else\quad d_{y} = 1;$$

Considering *x*_*s*_ as *x*_*s*_ + *d*_*x*_, continue the following process step through *x*_*s*_ + *d*_*x*_ and *n* − 1 times and until *x*_*s*_ becomes *x*_*e*_.

16 $$R[n][\theta ] = \left[ {x_{s} } \right]\left[ {m*x_{s} + b} \right]$$

If the absolute value of *d*_*x*_ is not larger than *d*_*y*_ and the value of *d*_*y*_ is not equal to 0, the point *R*[*n*][*θ*] from the slope *m* is computed as follows using the Eqs. (17) through (20).

17 $$m = \frac{{d_{x} }}{{d_{y} }}$$

The b (*y* intercept) is computed as

18 $$b = x_{s} - m*y_{s}$$

19 $$If\left( {d_{y} < 0} \right)\,\,then,\,\;d_{x} = - 1;\;\;else\;\;d_{y} = 1;$$

Considering *y*_*s*_ as *y*_*s*_ + *d*_*x*_, continue the following process step through *y*_*s*_ + *d*_*x*_ and *n* − 1 times and until *y*_*s*_ becomes *y*_*e*_.

20 $$R[n][\theta ] = \left[ {m*x_{s} + b} \right]\left[ {y_{s} } \right]$$

In this way, points from limbus border to pupil border for each position are traced and stored in angular resolution array with step $\phi$ times until *θ* becomes 360°.

The algorithm of the proposed variable-size normalization is given hereunder:
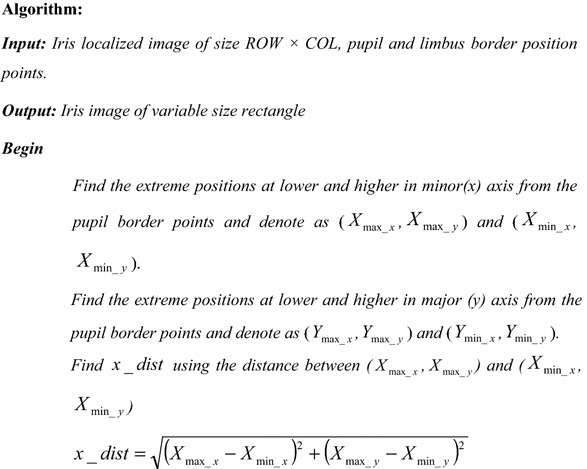

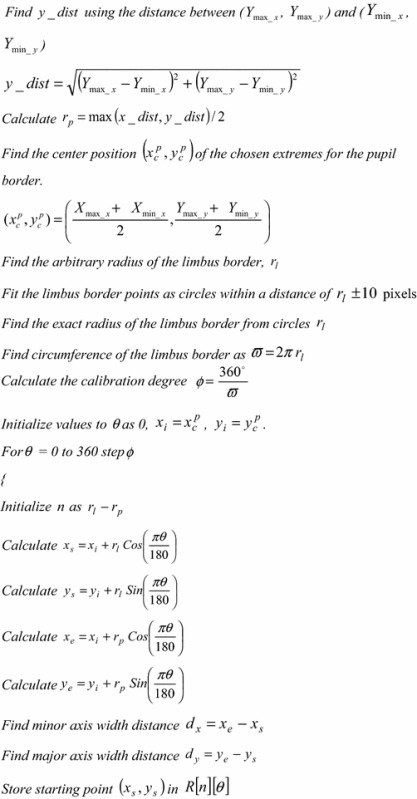

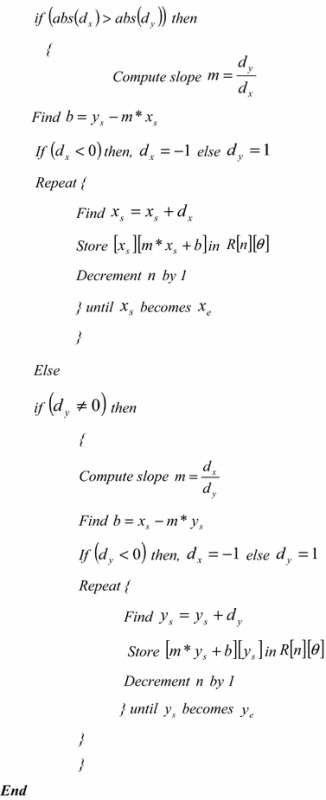


## Proposed fixed-size normalization model

First let us consider the pupil and limbus border points present in iris localized image. The radius of the pupil and the limbus border are computed as described in section “Proposed variable-size normalization model”. But in this fixed-size normalization model, the resolution angle of increment $\phi$ is in the interval 0–1. The maximum degree (360°) is divided into small units of angle increment factor $\phi$ that enables to reach more limbus border positions of the iris. Iris is normalized using pupil as the reference point. By increasing the angle *θ* by $\phi$ for radius *r*_*l*_, the fixed-size rectangular resolution iris image for the plane (*θ*, *r*_*l*_) is obtained. After fixing the resolution angle of increment, the sampling points are extracted by tracing points from pupil border to limbus border. Initialize *θ* as 0, *n* as 0, *x*_*i*_ as $x_{c}^{p}$, and *y*_*i*_ as $y_{c}^{p}$. Let the number of tracing points *n* from pupil border to limbus border with the length $\left( {r_{l} - r_{p} } \right)$. The starting point (*x*_*s*_, *y*_*s*_) of tracing process is computed as

21 $$x_{s} = x_{i} + r_{p} Cos\left( {\frac{\pi \theta }{180}} \right)$$

22 $$y_{s} = y_{i} + r_{p} Sin\left( {\frac{\pi \theta }{180}} \right)$$

The end point (*x*_*e*_, *y*_*e*_) of tracing process is computed as

23 $$x_{e} = x_{i} + r_{l} Cos\left( {\frac{\pi \theta }{180}} \right)$$

24 $$y_{e} = y_{i} + r_{l} Sin\left( {\frac{\pi \theta }{180}} \right)$$

In this way, points from pupil border to limbus border for each position are traced and stored in angular resolution array step $\phi$ times until *θ* becomes 360°.

The algorithm of the proposed fixed-size normalization is given hereunder:
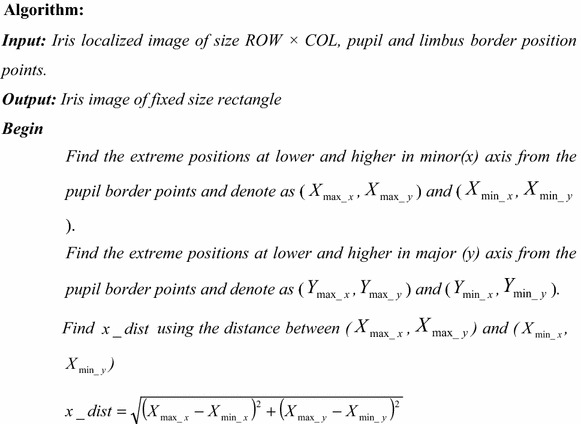

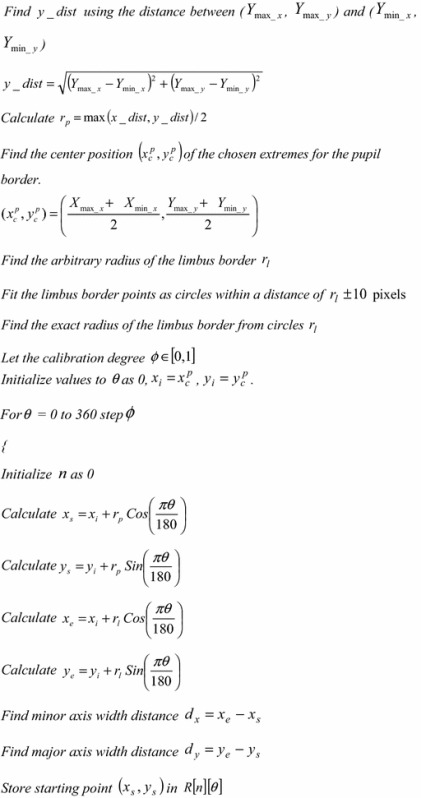

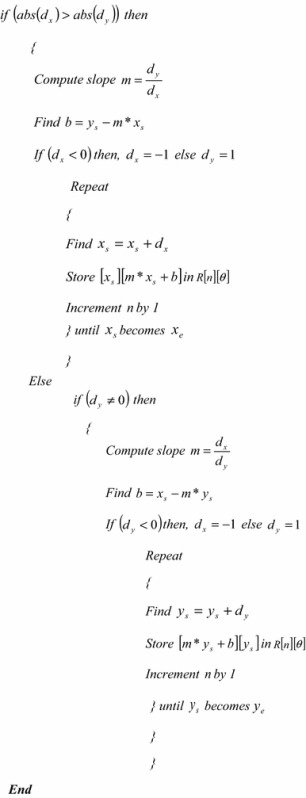


## Orthogonal polynomials based iris recognition

With a view to extract iris texture feature, the normalized iris is further subjected to the orthogonal polynomials to extract the transformed coefficients (Ganesan and Bhattacharya 1997). The variance is computed from the transformed coefficients and the sets such as main effects, interaction effects are obtained (Krishnamoorthi and Kannan 2009). The spatial variation that causes the interaction effects are owing to micro texture present in the iris region. To investigate whether a specified region possesses texture characteristics, the Hartley’s criteria are applied for testing the homogeneity amongst variances (Krishnamoorthi and Anna Poorani 2012). Once, texture regions are identified, the F-ratio test is applied for computing the SNR and the result of the F-ratio test for determining significance towards the micro texture is encoded as a binary string. The corresponding decimal numeral is found subsequently to characterise the micro texture (Krishnamoorthi et al. 2013). The numerical characterization sequence is used as feature vector for further processing in iris recognition.

The dimension of features in feature vector is reduced by means of LDA (Liu and Xie 2006). It is employed to discard the null space of between_class_scatter *S*_*b*_, by first diagonalizing between_class_scatter *S*_*b*_ and then diagonalizing within_class_scatter *S*_*w*_. The support vectors of the query image are computed to match the query image with the database images from the reduced feature vector using Nonlinear asymmetrical support vector machine (SVM) matching scheme (Roy and Bhattacharya 2006).

## Empirical results and discussion

**Fig. 2:**
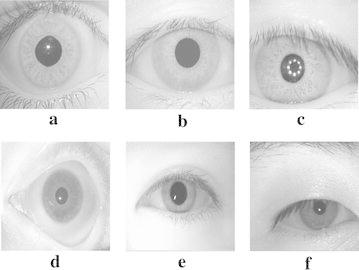
Sample test images considered for iris normalization from different databases such as **a** BITIRIS database, **b** CASIA V 1.0, **c** CASIA V 3.0 Interval, **d** BATH, **e** MMU V 1.0, **f** MMU V 2.0

The proposed normalization techniques have been experimented with the BITIRIS Database (2013) and existing standard iris databases. CASIA Database V 1.0 and V 3.0 Interval (2013), Bath Database (2013) and MMU Database V 1.0 and V 2.0 (2013) are also exploited in experiments. Few sample images from these databases, used for normalization experiments are shown in Fig. 2.

**Fig. 3:**
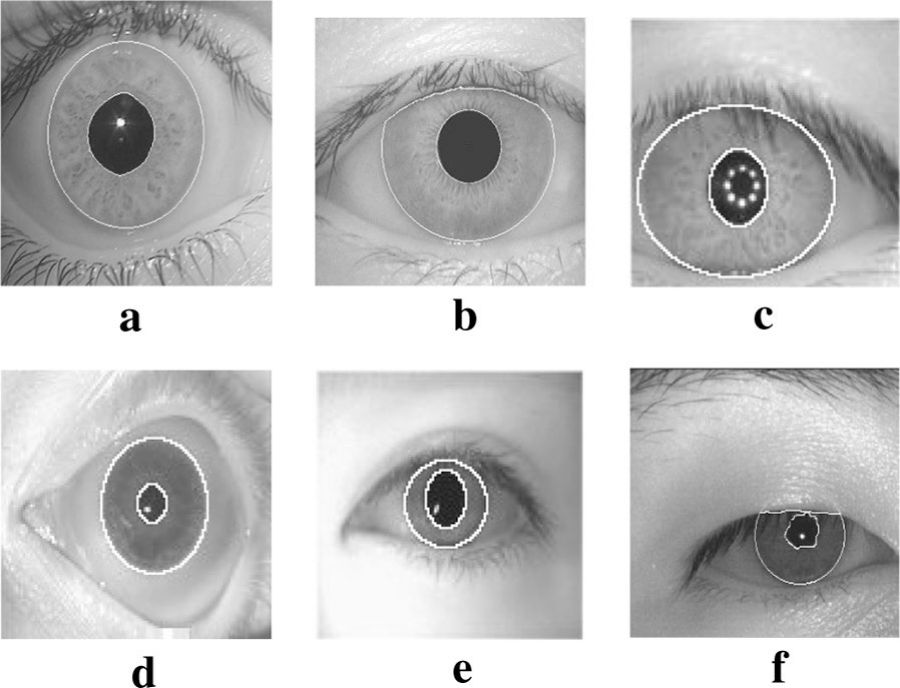
Outcomes of iris localization. **a** BITIRIS. **b** CASIA Version 1.0. **c** CASIA Version 3.0 Interval. **d** BATH. **e** MMU Version 1.0. **f** MMU Version 2.0

**Fig. 4:**
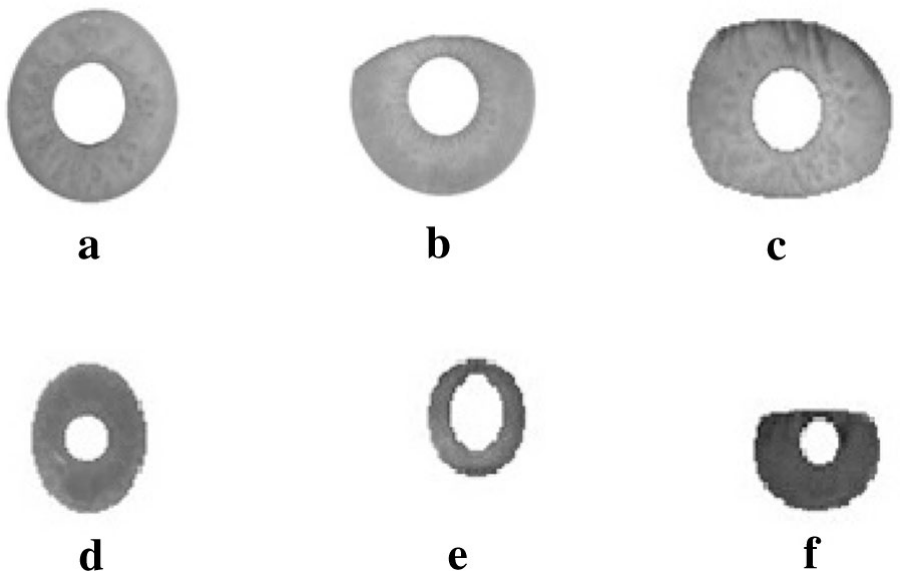
Outcomes of segmented iris image. **a** BITIRIS. **b** CASIA Version 1.0. **c** CASIA Version 3.0 Interval. **d** BATH. **e** MMU Version 1.0. **f** MMU Version 2.0

**Fig. 5:**
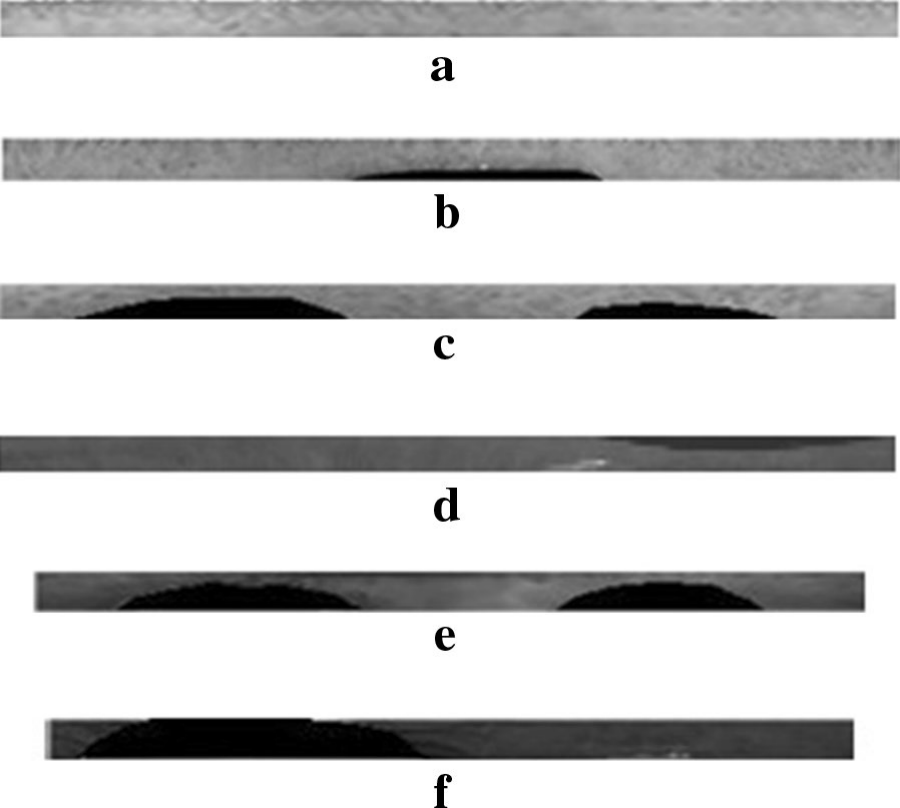
Outcomes of proposed variable size normalization. **a** BITIRIS. **b** CASIA Version 1.0. **c** CASIA Version 3.0 Interval. **d** BATH. **e** MMU Version 1.0. **f** MMU Version 2.0

Initially, the input images are preprocessed as described in section “Preprocessing”. The corresponding resultant iris localized images for test images shown in Fig. 2a–f, after iris localization process with orthogonal polynomials, are presented in Fig. 3a–f. The result of the iris segmentation corresponding to the pre-processed images are presented in Fig. 4a–f. The iris segmented image is subjected to the proposed variable-size normalization scheme as depicted in section “Proposed variable-size normalization model”. The approximate radius of pupil border is calculated from the radius of irregular pupil border. After determining the radius of limbus border points, the sampling points are found by tracing points from limbus border to pupil border using the coordinate conversion. The corresponding normalization result for the images shown in Fig. 2a–f, are presented in Fig. 5a–f.

**Fig. 6:**
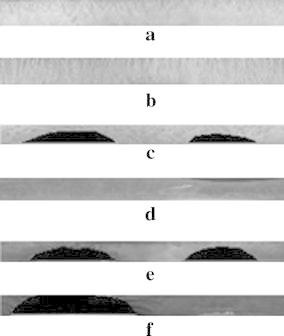
Outcomes of proposed fixed size normalization. **a** BITIRIS. **b** CASIA Version 1.0. **c** CASIA Version 3.0 Interval. **d** BATH. **e** MMU Version 1.0. **f** MMU Version 2.0

Similarly, the iris localized eye image is subjected to the proposed fixed-size normalization scheme as depicted in section “Proposed fixed-size normalization model”. The sampling points are found by tracing points from pupil border to limbus border in a fixed fashion. The corresponding normalization outcomes for the images shown in Fig. 2a–f, after normalization process with the proposed fixed size normalization scheme are presented in Fig. 6a–f. It is viewed from the Fig. 6 that the fixed-size model tends to produce fixed texture information. In this experiment, the calibration degree $\phi$ is varied and its impact in the overall precision of iris recognition is analyzed. It is observed that when $\phi$ < 0.6 there is a strong raise in the recognition error rate, stimulated as a result of aliasing taken place in the normalization process.

The performance of the proposed algorithms is calculated for BITIRIS with computation time intended for the normalization process. For this purpose, the proposed experiments are conducted on a Intel (R) core (TM) i7 CPU 965@3.20 GHz system with 4.00 GB RAM. These outcomes are obtained and tabulated in Table 1. From the Table 1, it is apparent that, the proposed variable-size normalization model takes more computation time when compared with the proposed fixed-size normalization model. The proposed variable-size normalization has positive impact on the feature extraction stage due to the presence of more texture. However the suitability of the proposed variable-size normalization model and proposed fixed-size normalization model for feature extraction, matching, etc. requires rigorous experimentation.

**Table 1:** Computation time for normalization process, with the proposed scheme on BITIRIS database images

Method used	Time taken for normalization (s)
Proposed variable size normalization model	2.234
Proposed fixed size normalization model	1.586

For an unbiased comparison, the orthogonal polynomials based iris recognition has been experimenting with the proposed variable-size normalization model and proposed fixed-size normalization model using images in BITIRIS database. For recognition, a query image was matched against the entire database of stored iris representations. The results of the matching process yield the highest similarity across the registered images for each class were chosen as the matching iris. The performance of the proposed variable-size normalization model and fixed-size normalization models are evaluated with orthogonal polynomials based iris recognition system in terms of standard FAR, FRR and CRR. The empirical outcomes of the proposed variable size normalization for the identification with FAR, FRR and CRR are tabulated in Table 2. A recognition performance of FAR = 0.010 %, FRR = 0.112 % and CRR = 99.88 % is obtained with the proposed variable size normalization model with orthogonal polynomials based iris recognition system. A recognition performance of FAR = 0.015 %, FRR = 0.165 % and CRR = 99.82 % is also obtained with the proposed fixed size normalization model with orthogonal polynomials based iris recognition system.

**Table 2:** Outcomes of proposed iris normalization schemes on BITIRIS database images in terms of FAR, FRR and CRR

Method	BITIRIS database		
	FAR (%)	FRR (%)	CRR (%)
Proposed variable size normalization model + orthogonal polynomials based iris recognition system	0.010	0.112	99.88
Proposed fixed size normalization model + orthogonal polynomials based iris recognition system	0.015	0.165	99.82

It is clear from Table 2, that the proposed variable-size normalization scheme outperforms the proposed fixed-size normalization scheme.

**Fig. 7:**
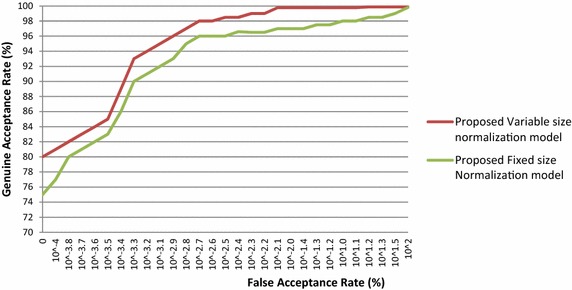
ROC curve of proposed iris normalization schemes on the BITIRIS database images

For verification, ROC curves are drawn by plotting the GAR as a function of the FAR in semi-logarithmetic scale. The ROC curves of proposed variable-size normalization model and proposed fixed-size normalization model are plotted for the BITIRIS database and these outcomes are presented in Fig. 7.

It is exemplified from the Fig. 7 that the proposed variable size iris normalization scheme attains higher GAR with an extremely low EER than fixed size iris normalization scheme on the BITIRIS database.

The EER of the proposed variable-size normalization model and proposed fixed-size normalization model is observed from the ROC curve and are tabulated in the Table 3. The EER of the proposed variable size normalization is only 0.100 % as against 0.145 % for fixed-size normalization model on the BITIRIS database.

**Table 3:** Outcomes of proposed iris normalization schemes on BITIRIS database images in terms of EER

Method	EER (%)
Proposed variable size normalization model + orthogonal polynomials based iris recognition system	0.100
Proposed fixed size normalization model + orthogonal polynomials based iris recognition system	0.145

It is evident from Table 3 that the EER of the variable size iris normalization scheme is found to be superior than fixed size iris normalization scheme. It is also well-known from Table 3 that the proposed variable size normalization scheme is able to attain close proximity to zero EER. Extremely low EER of 0.100 % reveals the robustness of the variable size iris normalization scheme in verification mode.

**Fig. 8:**
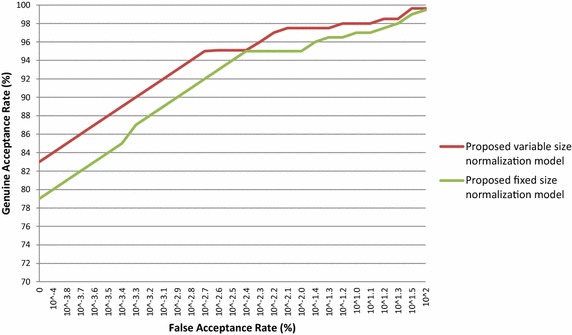
ROC curve of proposed iris normalization schemes on the CASIA V 1.0 database images

**Fig. 9:**
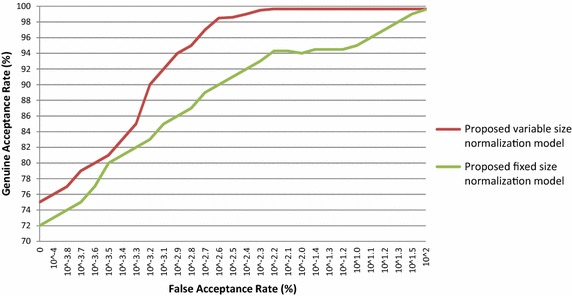
ROC curve of proposed iris normalization schemes on the CASIA V 3.0 Interval database images

**Fig. 10:**
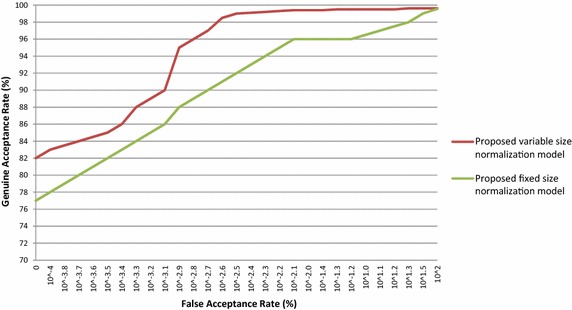
ROC curve of proposed iris normalization schemes on the BATH database images

**Fig. 11:**
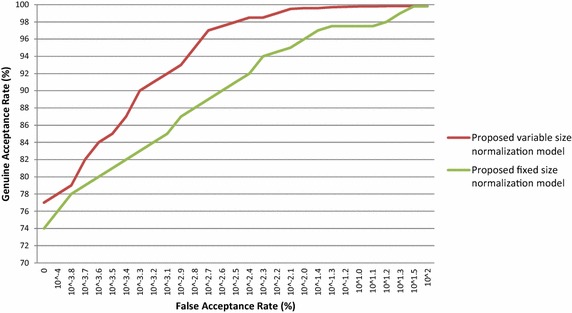
ROC curve of proposed iris normalization schemes on the MMU V 1.0 database images

**Fig. 12:**
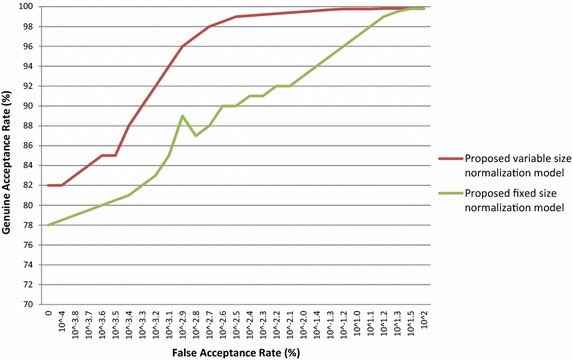
ROC curve of proposed iris normalization schemes on the MMU V 2.0 database images

Similarly, the proposed iris normalization schemes are also used to authenticate the person in other database images viz CASIA V 1.0, CASIA V 3.0 Interval, BATH, MMU V 1.0 and MMU V 2.0. The performance of the proposed iris normalization schemes is evaluated with standard FAR, FRR and CRR for various database images and their outcomes are tabulated in Table 4. The ROC curves are plotted for the above mentioned database and these outcomes are presented in Figs. 8, 9, 10, 11 and 12. The EER of the iris normalization schemes are observed from the ROC curve and are tabulated in the Table 5. It is inferred from the empirical outcomes that the proposed variable size normalization model outperforms proposed fixed size normalization model, showing their robustness across different imaging environments.

**Table 4:** Outcomes of proposed iris normalization schemes on various iris database images in terms of FAR, FRR and CRR

Iris data base	Method	FAR (%)	FRR (%)	CRR (%)
CASIA V 1.0	Proposed variable size normalization model + orthogonal polynomials based iris recognition system	0.011	0.354	99.635
	Proposed fixed size normalization model + orthogonal polynomials based iris recognition system	0.013	0.523	99.464
CASIA V 3.0 Interval	Proposed variable size normalization model + orthogonal polynomials based iris recognition system	0.021	0.312	99.667
	Proposed fixed size normalization model + orthogonal polynomials based iris recognition system	0.025	0.399	99.576
BATH	Proposed variable size normalization model + orthogonal polynomials based iris recognition system	0.032	0.343	99.625
	Proposed fixed size normalization model + orthogonal polynomials based iris recognition system	0.037	0.409	99.554
MMU V 1.0	Proposed variable size normalization model + orthogonal polynomials based iris recognition system	0.019	0.156	99.825
	Proposed fixed size normalization model + orthogonal polynomials based iris recognition system	0.021	0.171	99.808
MMU V 2.0	Proposed variable size normalization model + orthogonal polynomials based iris recognition system	0.031	0.16	99.809
	Proposed fixed size normalization model + orthogonal polynomials based iris recognition system	0.032	0.171	99.797

**Table 5:** Outcomes of proposed iris normalization schemes on various iris database images in terms of EER

Iris database	Method	EER (%)
CASIA V 1.0	Proposed variable size normalization model + orthogonal polynomials based iris recognition system	0.591
	Proposed fixed size normalization model + orthogonal polynomials based iris recognition system	0.651
CASIA V 3.0 Interval	Proposed variable size normalization model + orthogonal polynomials based iris recognition system	1.032
	Proposed fixed size normalization model + orthogonal polynomials based iris recognition system	1.151
BATH	Proposed variable size normalization model + orthogonal polynomials based iris recognition system	1.014
	Proposed fixed size normalization model + orthogonal polynomials based iris recognition system	1.214
MMU V 1.0	Proposed variable size normalization model + orthogonal polynomials based iris recognition system	0.156
	Proposed fixed size normalization model + orthogonal polynomials based iris recognition system	0.161
MMU V 2.0	Proposed variable size normalization model + orthogonal polynomials based iris recognition system	0.0007
	Proposed fixed size normalization model + orthogonal polynomials based iris recognition system	0.0009

## Conclusion

In this paper, two different normalization methods are proposed that compensate the change in size of the iris due to the action of stretching or enlarging the pupil in iris acquisition process and camera to eyeball distance. In the first method, the variable dimension is used for irregular iris images to avoid under the samples near the limbus border. In the second method, the fixed dimension is used for irregular iris images with a rectangular model to circumvent the dimensional discrepancies among the iris images. The proposed normalization methods are compared along with the orthogonal polynomials based iris recognition and analyzed to enhance the normalization stage. The impacts of proposed variable-size normalization versus rectangular normalization on extracted features are presented. From the empirical outcomes, it is examined that the variable-size normalization scheme performs better than the fixed-size normalization approach in terms of matching. It is concluded that the proposed variable-size normalization makes orthogonal polynomials based iris recognition system more robust to the illumination variations than the proposed fixed-size normalization model.
